# Understanding the effects of cortical gyrification in tACS: insights from experiments and computational models

**DOI:** 10.3389/fnins.2023.1223950

**Published:** 2023-08-16

**Authors:** Jesús Cabrera-Álvarez, Jaime Sánchez-Claros, Martín Carrasco-Gómez, Alberto del Cerro-León, Carlos J. Gómez-Ariza, Fernando Maestú, Claudio R. Mirasso, Gianluca Susi

**Affiliations:** ^1^Centre for Cognitive and Computational Neuroscience, Complutense University of Madrid, Madrid, Spain; ^2^Department of Experimental Psychology, Complutense University of Madrid, Madrid, Spain; ^3^Instituto de Física Interdisciplinar y Sistemas Complejos (IFISC, UIB-CSIC), Campus UIB, Palma de Mallorca, Spain; ^4^Biomedical Image Technologies, ETSI Telecomunicación, Universidad Politécnica de Madrid, Madrid, Spain; ^5^Department of Psychology, University of Jaén, Jaén, Spain; ^6^Department of Structure of Matter, Thermal Physics and Electronics, School of Physics, Complutense University of Madrid, Madrid, Spain

**Keywords:** tACS, spiking neural networks, brain network models, MEG, neuromodulation

## Abstract

The alpha rhythm is often associated with relaxed wakefulness or idling and is altered by various factors. Abnormalities in the alpha rhythm have been linked to several neurological and psychiatric disorders, including Alzheimer's disease. Transcranial alternating current stimulation (tACS) has been proposed as a potential tool to restore a disrupted alpha rhythm in the brain by stimulating at the individual alpha frequency (IAF), although some research has produced contradictory results. In this study, we applied an IAF-tACS protocol over parieto-occipital areas to a sample of healthy subjects and measured its effects over the power spectra. Additionally, we used computational models to get a deeper understanding of the results observed in the experiment. Both experimental and numerical results showed an increase in alpha power of 8.02% with respect to the sham condition in a widespread set of regions in the cortex, excluding some expected parietal regions. This result could be partially explained by taking into account the orientation of the electric field with respect to the columnar structures of the cortex, showing that the gyrification in parietal regions could generate effects in opposite directions (hyper-/depolarization) at the same time in specific brain regions. Additionally, we used a network model of spiking neuronal populations to explore the effects that these opposite polarities could have on neural activity, and we found that the best predictor of alpha power was the average of the normal components of the electric field. To sum up, our study sheds light on the mechanisms underlying tACS brain activity modulation, using both empirical and computational approaches. Non-invasive brain stimulation techniques hold promise for treating brain disorders, but further research is needed to fully understand and control their effects on brain dynamics and cognition. Our findings contribute to this growing body of research and provide a foundation for future studies aimed at optimizing the use of non-invasive brain stimulation in clinical settings.

## 1. Introduction

The oscillatory behavior of the brain is characterized by a well-defined set of rhythms that play a crucial role in cognitive processes such as attention, memory and perception, among others. Importantly, the alpha rhythm, which typically ranges between 8 and 12 Hz and is prominent in posterior regions of the brain (Britton et al., 2016), is often associated with a state of relaxed wakefulness or idling (Buzsaki, 2006) and can be altered by various factors such as sensory stimulation, mental effort, and attention (Webster and Ro, 2020). It has been suggested that the alpha rhythm reflects inhibitory processes that suppress irrelevant sensory inputs and promote the processing of internal information (Payne and Sekuler, 2014). Abnormalities in the alpha rhythm have been associated with various neurological and psychiatric disorders (see, e.g., Babiloni et al., 2020; Ippolito et al., 2022). For instance, in Alzheimer's disease, a reduction in alpha power relative to healthy controls has been reported along the disease progression (Babiloni et al., 2009; López-Sanz et al., 2016; Lejko et al., 2020). These previous findings raise up the question, whether the modulation of those brain rhythms could improve cognitive functions in these clinical conditions or even in control subjects.

Transcranial alternating current stimulation (tACS) is a non-invasive brain stimulation technique that uses external electrical currents to modulate the neuronal oscillatory activity at specific frequencies. Similar to other types of brain stimulation, tACS has the potential to advance our understanding of brain function by establishing causal links between brain activity, cognition, and behavior (Dayan et al., 2013; Herrmann et al., 2013; Polańıa et al., 2018). The mechanistic underpinnings of tACS imply weak electrical currents delivered through the skull altering the polarization of cellular membranes, and thereby modulating the thresholds of neural activation (Reato et al., 2013; Liu et al., 2018). This effect can enhance neural synchronization by biasing the timings of neural activation with the rhythm of the stimulation (Helfrich et al., 2014; Liu et al., 2018; Vogeti et al., 2022). Additionally, some recent reports point out that tACS may also modulate neural plasticity, inducing long-term potentiation and depression (Jeong et al., 2021; Schwab et al., 2021).

tACS has emerged as a promising technique for modulating brain activity and has shown promise in reversing the reduction of alpha power observed in several disorders. Some studies have already explored the use of a specific application of tACS at the individual alpha frequency (IAF) to enhance the power of alpha oscillations (Zaehle et al., 2010; Helfrich et al., 2014; Kasten et al., 2019; Zarubin et al., 2020). In this type of experiments, the IAF-tACS stimulation is delivered at parieto-occipital regions to generate an entrainment with the predominant posterior alpha rhythm. The results of these experiments are contradictory, both in terms of the effective results of stimulation, and the locations where the effects are found. tACS can be influenced by several factors, including the selection of electrode montages, current dosage, targeted regions, skull conductivity, head positioning, cortex morphology, and cells' orientation (Datta et al., 2012; Huang et al., 2019; Kasten et al., 2019; Guerra et al., 2020).

One of the issues being discussed is how the orientation of the cellular body axis influences the effects of electric field stimulation. Pyramidal neurons are arranged in the form of a palisade, with their main axes parallel to each other and perpendicular to the cortical surface (Hansen et al., 2010; Susi et al., 2019; da Silva, 2022). Their elongated morphology makes them responsive to the application of external electromagnetic fields (Liu et al., 2018; Aberra et al., 2020), and previous research has demonstrated that the impact of the electric fields depends on its relative orientation with respect to the axodendritic direction of the pyramidal cells' bodies (Radman et al., 2009; Dmochowski et al., 2012; Aberra et al., 2020). However, more research is needed to optimize tACS and better understand the variables that affect its mechanisms. This will pave the way for efficient and controlled tACS application, leading to better outcomes for patients.

Computational modeling has emerged as a valuable tool for investigating the mechanistic effects of electrical stimulation on the brain. Within this field of research, two types of models have become particularly prominent: current propagation models and neural activation models. Current propagation models are designed to analyze, predict, and regulate the electric fields produced in the brain by the applied stimulation. These models consider the impact of different head tissues, their shape, and the specific montages used for stimulation (Holdefer et al., 2006; Miranda et al., 2006; Russell et al., 2014; Huang et al., 2017; Forssell et al., 2021). Neural activation models provide a detailed understanding of how electric fields generated by stimulation impact the activation of neural tissue at different levels of analysis, ranging from individual cells to the entire brain (Merlet et al., 2013; Deco et al., 2019; Aberra et al., 2020; Meier et al., 2022; Tran et al., 2022; Wang et al., 2023). These models offer a more comprehensive explanation of the effects of stimulation. Neural activation models can be integrated into a network of brain regions (i.e., *brain network model*), and stimulated *in-silico* (Merlet et al., 2013). To ensure the accuracy of the models, they are usually calibrated using empirical data to replicate the phenomenon being studied as closely as possible. By combining empirical and computational methodologies, researchers can strengthen their understanding of the mechanisms underlying electrical stimulation and the effects on brain function.

Our study utilized a bipolar electrode montage to deliver alternating currents to the posterior regions of the brain to modulate alpha power through an IAF-tACS stimulation protocol. Given the literature mentioned before, we hypothesized to achieve an increase in alpha power over parieto-occipital areas. To gain a deeper understanding of the results and explain any variability observed in them, we constructed and analyzed a spiking neural network (SNN) model comprising neural populations distributed throughout large brain networks, hypothesizing that cortical gyrification can be used to explain the effects of neurostimulation. Our findings provide insights that could facilitate more precise and effective application of tACS in the future.

## 2. Methods

### 2.1. Empirical methods

#### 2.1.1. Study design

The study consisted of one tACS session and three magnetoencephalography (MEG) scans, two before (i.e., *pre1* and *pre2* sessions) and one after the stimulation (*post* session). We initially captured the neurophysiological activity of each participant through two successive 5-min eyes-closed resting state MEG recordings, with a 10-min interval between sessions. The reason to include two MEG recordings before the stimulation was to account for any possible variability in the individual alpha-peak frequency (IAF) of the participants. The IAF was derived from each recording using a fast preprocessing algorithm that we describe in a later section, and then averaged. Participants were then randomly assigned to either the *verum* or *sham* group, and received 20 min of stimulation at their own IAF with their eyes closed. Immediately after the stimulation, we performed a third MEG recording to measure its effects on the participants. We decided to use a resting-state eyes-closed paradigm given its prominent alpha activity, and previous work on the same kind of stimulation (Zarubin et al., 2020; Wang et al., 2022).

#### 2.1.2. Empirical dataset

Eleven female and 16 male healthy participants, with ages ranging from 22 to 55 years (32.80 ± 8.52 and 32.00 ± 8.98, respectively) were recruited at the Center for Cognitive and Computational Neuroscience (C3N) associated with the Complutense University of Madrid (UCM) for the neurostimulation study. Our study included only right-handed, native Spanish-speaking participants without any previous neuropsychiatric history or metallic prostheses that could interfere with neuroimaging and neuromodulation. Participants with undistinguishable IAF were also excluded. We followed current guidelines and safety regulations throughout the research, and obtained informed consent from every participant before their participation.

MEG signals were acquired during 5 min of eyes-closed resting state at 1 kHz sampling rate, using 306 channels (102 magnetometers and 204 gradiometers) whole-head Elekta Neuromag system (Elekta AB, Stockholm, Sweden) located in a magnetically isolated room (VacuumSchmelze GmbH, Hanau, Germany). Using a Fastrak 3D digitizer (Polhemus, Colchester, Vermont), the positions of four head position indicator (HPI) coils attached to the scalp were defined and the shape of each participant's head relative to three anatomical locations (nasion and both preauricular points) was modeled. An online anti-aliasing filter [(0.1–330) Hz] was applied during the whole session.

Raw data was pre-processed by the Maxfilter software (v.2.2, correlation threshold = 0.9, time window = 10 s) to remove the environmental noise using the temporal extension of the signal space separation (tSSS) method with movement compensation (Taulu and Simola, 2006). Given the elevated redundancy of gradiometer data after applying the tSSS (Garcés et al., 2017) only data from magnetometers were considered for subsequent analyzes. Eye, muscle, and jump artifacts were automatically located using Fieldtrip software (Oostenveld et al., 2011) and reviewed by MEG signal experts. Finally, the noise-free signal was divided into 4-second segments and an independent component analysis (ICA) based on SOBI (Belouchrani et al., 1997) was used to remove eye-blink and cardiac magnetic field artifacts. After applying ICA, we eliminated all segments that still contained any eye-blink or muscle artifacts. Preprocessed MEG data was then used to carry out source localization using a Linearly Constrained Minimum Variance (LCMV) beam former (Van Veen and Buckley, 1988). Because we did not have a T1 MRI for all subjects, a 1 mm resolution template of healthy adults normalized to the Montreal Neurological Institute (MNI) 1 mm voxel size template was used to place the sources inside the brain in a homogeneous grid of 1 cm. Then both the template and grid were linearly transformed to fit the head shape of each subject. The lead fields were defined using a local spheres approach to fit the head shape of each subject in the vicinity of each sensor. Spatial filter coefficients were estimated for each subject using the computed lead field and an average of the covariance matrix for all the segments. Thereafter, this filter was used to compute the source time series separately for each segment and source location. Sources were grouped according to the Automated Anatomical Labeling (AAL) atlas cortical map (Tzourio-Mazoyer et al., 2002). From the original 4,560 source locations, only those 2,459 labeled as belonging to an area defined in the atlas were considered in the following steps. The other sources were placed in areas not defined in the atlas (i.e., white matter, CSF, or subcortical regions) and therefore cannot be source generators of MEG signals (Hämäläinen et al., 1993). From the reconstructed activity, the power spectrum for each cortical source was calculated by applying a multi-tapering method of Slepian sequences (DPSS) (Slepian, 1978; McCoy et al., 1998) between 2 and 45 Hz, then normalizing by dividing the spectra by the power between 2 and 45 Hz. The individual alpha frequency (IAF) was determined using a fast-processing algorithm on the *pre1* and *pre2* session scans, immediately after their recording. The data was processed using the same pipeline as described in the previous paragraph, but some steps were omitted to obtain a quick result, as the resulting frequency was needed for subsequent stimulation of the participant. Specifically, no tSSS or movement compensation method was applied, and manual revision of artifacts was skipped as well. Independent component analysis (ICA) was used to remove eye-blink and cardiac activity, while the remaining contaminated segments were removed manually. The power spectrum for each magnetometer was calculated using DPSS, and then averaged. The resulting spectra were visually inspected, and the frequency of the power peak in the alpha band (8–12 Hz) was defined as the IAF. The IAFs from sessions *pre1* and *pre2* were averaged to determine the final frequency for the neurostimulation procedure.

#### 2.1.3. Neurostimulation

The neurostimulation sessions were carried out using a bipolar tACS stimulation with two conductive rubber electrodes (7 × 5 cm) located at Cz and Oz (midline central and midline occipital, respectively) using a microprocessor-controlled current source *NeuroConn DC-StimulatorPlus* (Neurocare, Ilmenau, Germany). The electrodes were covered by sponges and wet with saline solution. Verum stimulation was performed at the IAF during a 20-min session with a current intensity of 3 mA peak-to-peak, while those undergoing sham stimulation only received stimulation during the fade-in and fade-out periods (30 s each).

#### 2.1.4. Statistical analysis

To assess the effect of stimulation in areas with maximal current density, an independent sample t-test was conducted to compare the pre2-post rate of change in the power of the IAF (±2 Hz bandwidth) between the *verum* and *sham* groups. The statistical analysis focused on the parieto-occipital regions of the AAL atlas, including the Calcarine fissure, Cuneus, Occipital lobe, Parietal gyrus, Angular gyrus, Precuneus, and Paracentral lobule. The statistical test was right-tailed since we hypothesized that the *verum* group would experience an increase in power due to the neurostimulation session. We chose to use the *pre2* MEG recording as a baseline because it was the closest to the neurostimulation session, and all patients had experienced a similar situation until that point. Furthermore, we utilized a non-parametric cluster-based permutation (CBPT) test (Bullmore et al., 1999) to investigate the *pre2*-*post* changes in power. This analysis enabled us to identify significant differences at the source level without the need for atlases and frequency bands and accounted for the problem of multiple comparisons. To align the IAFs of each participant, we shifted the spectra of each individual. An analysis of variance (ANCOVA) was conducted at each node, including age and sex as covariates, and the source-level significance threshold and cluster-level significance threshold were set at 0.05. Finally, we performed a Levene test, again on the parieto-occipital regions in the AAL atlas, averaging the power over the frequencies in which the previous CBPT analysis revealed significant changes. This analysis aimed to examine the variability of the effects of tACS.

### 2.2. Computational methods

#### 2.2.1. Data for simulations

For the computational modeling, a different set of data was needed, as MRI-T1 or dw-MRI recordings from the participants in the empirical dataset were not available. We used a dataset owned by the C3N consisting of 10 healthy subjects (seven females; age 69 ± 4.16) with resting-state eyes-closed MEG, MRI-T1 and dw-MRI recordings. MEG acquisition and preprocessing were performed following the description in the previous section. T1-MRIs were recorded using a General Electric 1.5 Tesla magnetic resonance scanner, using a high-resolution antenna and a homogenization PURE filter (fast spoiled gradient echo sequence, with parameters: repetition time/echo time/inversion time = 11.2/4.2/450 ms; flip angle = 12°; slice thickness = 1 mm, 256 × 256 matrix, and field of view = 256 mm). dw-MRIs were acquired with a single-shot echo-planar imaging sequence with the parameters: echo time/repetition time = 96.1/12,000 ms; NEX 3 for increasing the SNR; slice thickness = 2.4 mm, 128 × 128 matrix, and field of view = 30.7 cm yielding an isotropic voxel of 2.4 mm; 1 image with no diffusion sensitization (i.e., T2-weighted b0 images) and 25 dw-MRI (b = 900s/mm^2^).

To obtain the functional connectivity (FC) matrices, source reconstruction was performed using the minimum norm estimates method (Hämäläinen and Ilmoniemi, 1994), with the *constrained dipoles* variant, by which the current dipoles are oriented perpendicular to the cortical surface, to model the orientation of the macrocolumns of pyramidal neurons (Tadel et al., 2019). Source-space signals were then filtered in the alpha (8–12 Hz) band to calculate functional connectivity between sources using the Phase Locking Value (PLV, Lachaux et al., 1999). For the calculation of PLV, the instantaneous phase of each time-series is given by the Hilbert transform at time points *t* in every segment *n*, and then the following equation is applied:


(1) $$\begin{array}{l} PLV=\frac{1}{N}|\overset{N}{\underset{n=1}{\sum }}exp\left(j\phi \left(t,n\right)\right)| \end{array}$$

Where ϕ(*t, n*) is the phase difference of the two time series at time *t* and trial *n*. The resulting source matrices were averaged into AAL parcellation scheme.

The constrained dipoles method allowed us to obtain the source space signal at one point, taking into account the real orientation of the subtended cortical column, an aspect that is crucial to avoid sign/phase errors on the reconstructed time series. This is useful for computational purposes but recommended only when the T1-MRI of the participant is available.

To obtain structural connectivity (SC) matrices, a deterministic fiber tracking algorithm (Yeh et al., 2013) was used with augmented tracking strategies (Yeh, 2020) to improve reproducibility using DSI studio (http://dsi-studio.labsolver.org). The angular threshold was randomly selected from 15 to 90 degrees. Tracks with lengths shorter than 15 or longer than 180 mm were discarded. A total of 5 million seeds were placed in the whole brain volume. AAL atlas was used as the brain parcellation and the connectivity matrix was calculated by counting the number of connecting tracks passing through each pair of regions. Additionally, we extracted a mean track length for each pair of regions.

#### 2.2.2. Spiking population model

We built a spiking neural network with 22 regions extracted from the AAL atlas reproducing the cingulum bundle of the brain (Bubb et al., 2018), one of the most prominent white matter structures interconnecting frontal, parietal, and temporal lobes (Bubb et al., 2018). Each region was modeled as a balanced fully-connected network with 80 excitatory and 20 inhibitory neurons. The dynamics of the membrane potential of each neuron was described by the adaptive exponential integrate-and-fire (*aeif*) model (Naud et al., 2008), while the dynamics of the synapses was described by the alpha function. Both dynamics were implemented together in the *aeif_cond_alpha_multisynapse* class in the NEST environment package (Gewaltig and Diesmann, 2007; Eppler et al., 2008). Mathematically, the dynamics of the neuron *i* in the population *k* was described as follows:


(2) $$\begin{array}{l} C_{i}\frac{dv_{i}^{k}}{dt}=-g_{\text{L},i}(v_{i}^{k}-E_{\text{L},i})+g_{\text{L},i}\Delta_{T,i}\exp(\frac{v_{i}^{k}-v_{\text{th},i}}{\Delta_{T,i}})\\ \;-w_{i}^{k}+I_{i}^{k}+I_{\text{noise},i}^{k}-I_{\text{syn,i}}^{k}+I_{\text{ext},i}^{k},\\ \tau_{w,i}\frac{dw_{i}^{k}}{dt}=a_{i}(v_{i}^{k}-E_{\text{L},i})-w_{i}^{k},\\ \text{ if }v_{i}^{k}>v_{\text{vpeak}}\{\begin{array}{ll} v_{i}^{k} & \to v_{\text{reset},i}\\ w_{i}^{k} & \to w_{\text{reset},i}^{k}=w_{i}^{k}+b_{i} \end{array} \end{array}$$

where $I_{i}^{k}$ is an external bias current. $I_{noise,i}^{k}$ is a current generated by a Poissonian spike train with a rate of 2.4 kHz that accounted for the activity received from neurons that were not included in the population. $I_{syn,i}^{k}$ is the sum of all synaptic currents, and $I_{ext,i}^{k}$ is the current produced by an external sinusoidal stimulation. Parameters without superindex *k* mean that their values only depend on whether the neuron is excitatory (*i*∈[1, 80]) or inhibitory (*i*∈[81, 100]). The values of the whole set of parameters and their description can be found in Table 1. These values were selected to replicate the somatic dynamics of the regular spiking pattern of pyramidal cells and the fast-spiking pattern of interneurons in the cortex for the excitatory and inhibitory neurons in the model, respectively (Naud et al., 2008).

**Table 1:** SNN parameters used in simulations.

**Parameter**	**Value**		**Unit**	**Description**
	**Exc**	**Inh**		
*C*	104	59	pF	Capacity of the membrane
*v*_reset_	−53.0	−54.0	mV	Reset value for *v*_*m*_ after a spike
*E*_L_	−65.0	−62.0	mV	Leak reversal potential
*g*_L_	4.3	2.9	nS	Leak conductance
*a*	−0.8	1.8	ns	Subthreshold adaptation
*b*	65.0	61.0	pA	Spike-triggered adaptation
Δ*T*	0.8	3.0	mV	Slope factor
τ_*w*_	88.0	16.0	ms	Adaptation time constant
*v*_th_	−52.0	−42.0	mV	Spike initiation threshold
*v*_vpeak_	0.0		mV	Spike detection threshold
*t*_ref_	2.0		ms	Duration of the refractory period
*I*	Variable		pA	Constant external input current
ḡ_AMPA_	Variable		nS	Maximum conductance of the excitatory synapses
ḡ_GABA_	Variable		nS	Maximum conductance of the inhibitory synapses
ḡ_noise_	Variable		nS	Maximum conductance of background activity synapses (AMPA)
*E*_AMPA_	0.0		mV	AMPA Excitatory reversal potential
*E*_GABA_	−85.0		mV	GABA Inhibitory reversal potential
τ_syn, AMPA_	3.0		ms	Rise time of AMPA excitatory synaptic conductance
τ_syn, GABA_	3.2		ms	Rise time of GABA inhibitory synaptic conductance
δ^*kk*^	1.0		ms	Intra-synaptic delay due to axon length
δ^*kk*′^	range		ms	Inter-synaptic (*k*≠*k*′) delay due to axon length

The synaptic current was expressed as the sum of the synapses within each population (intra-connectivity) and the synapses between different populations (inter-connectivity). While intra-synapses could be both excitatory (AMPA) and inhibitory (GABA_A), external long-range synaptic projections were assumed only excitatory (AMPA):


(3) $$\begin{array}{l} I_{\text{syn,i}}^{k}=I_{\text{intra},i}^{k}+I_{\text{inter},j}^{k},\\ I_{\text{intra,i}}^{k}=\sum_{j=1}^{n_{e}}A_{ij}^{kk}g_{ij,\text{AMPA}}^{kk}(t-\delta_{ij}^{kk})(v_{i}^{k}-E_{\text{AMPA}})\\ \;+\sum_{j=n_{e}+1}^{n_{e}+n_{i}}B_{ij}^{kk}g_{ij,\text{GABA}}^{kk}(t-\delta_{ij}^{kk})(v_{i}^{k}-E_{\text{GABA}}),\\ I_{\text{inter,i}}^{k}=\omega \sum_{\begin{array}{c} k^{\prime }=1\\ k^{\prime }\neq k \end{array}}^{N}\sum_{j=1}^{n_{e}}A_{ij}^{kk^{\prime }}g_{ij\text{AMPA}}^{kk^{\prime }}(t-\delta_{ij}^{kk^{\prime }})(v_{i}^{k}-E_{\text{AMPA}}),\\ g_{ij,\text{syn}}^{kk^{\prime }}(t)={\bar{g}}_{ij,\text{syn}}^{kk^{\prime }}(\frac{t-t_{j}^{k^{\prime }*}}{\tau_{\text{syn}}})\exp(\frac{t-t_{j}^{k^{\prime }*}-\tau_{\text{syn}}}{\tau_{\text{syn}}}) \end{array}$$

where *A* and *B* are the connectivity matrices for excitatory and inhibitory projections, δ is the matrix representing the delays in the synaptic connections, ω is the coupling factor, ${\bar{g}}_{ij,\text{syn}}^{kk^{\prime }}$ is the maximum strength of the synapse between presynaptic neuron *j* from population *k*′ and postsynaptic neuron *i* from population *k*, and $t_{j}^{k^{\prime }*}$ is the time when the presynaptic neuron *j* in population *k*′ spiked.

The local field potential (LFP) in each node was computed as the sum of all synaptic currents in that node.

The development of the SNN models was carried out in Python, and all scripts are available in the following github: https://github.com/LCCN/Frontiers2023.

#### 2.2.3. *Working point* simulations

A common protocol to adjust the model to empirical observations consists in determining the optimal coupling factor for which the similarity between the experimental functional connectivity (eFC) and the functional connectivity resulting from the simulation (sFC) is maximized. The degree of similarity is typically determined by the Pearson correlation between the vectorized upper triangular matrices, and therefore, the best fit is the one that maximizes this correlation (Cabral et al., 2014; Nakagawa et al., 2014). However, we considered two additional conditions: on one hand, to avoid highly synchronized states in the SNN model, we discarded unrealistically high mean PLV values as a criterion to select the working point. To enable the exploration of alpha frequency bands relevant to our study, we selected working points where the main oscillatory frequency of the nodes fell within the alpha range (see Supplementary Figure 2). These simulations were performed three times during 25 s, removing the initial 4s to avoid transients.

#### 2.2.4. From the current propagation model to the stimulation of the SNN

**Figure 1:**
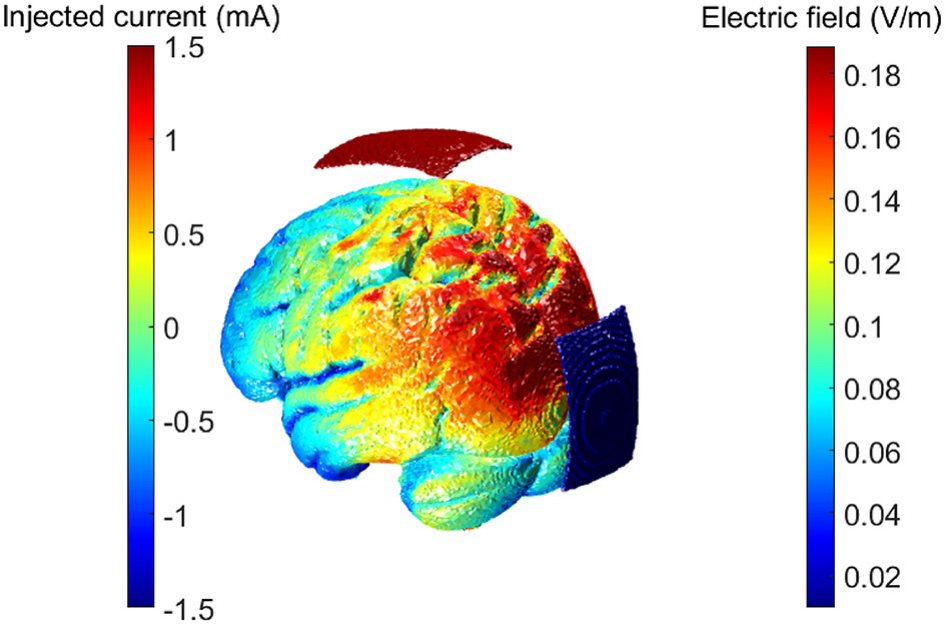
Electric field magnitudes computed with ROAST. The protocol used was the same as in the empirical experiment: Oz-Cz protocol. Left side colorbar referring to the injected current at the electrodes. Right side colorbar referring to the resulting electric field magnitude calculated for each cortical voxel.

We estimated the electric field generated in the brain with an Oz-Cz stimulation protocol (in line with the empirical experiment) using ROAST software (Huang et al., 2019). ROAST uses each of the participant's MRI-T1 images to segment brain tissues and generate a personalized FEM volumetric mesh. By assigning each tissue default values for conductivity and solving the underlying Laplace's equation, the software estimates the electric field propagation through the brain under DC stimulation (Huang et al., 2013, 2019). An electric field vector (in V/m) per MRI voxel is the main output of the model (see Figure 1).

**Figure 2:**
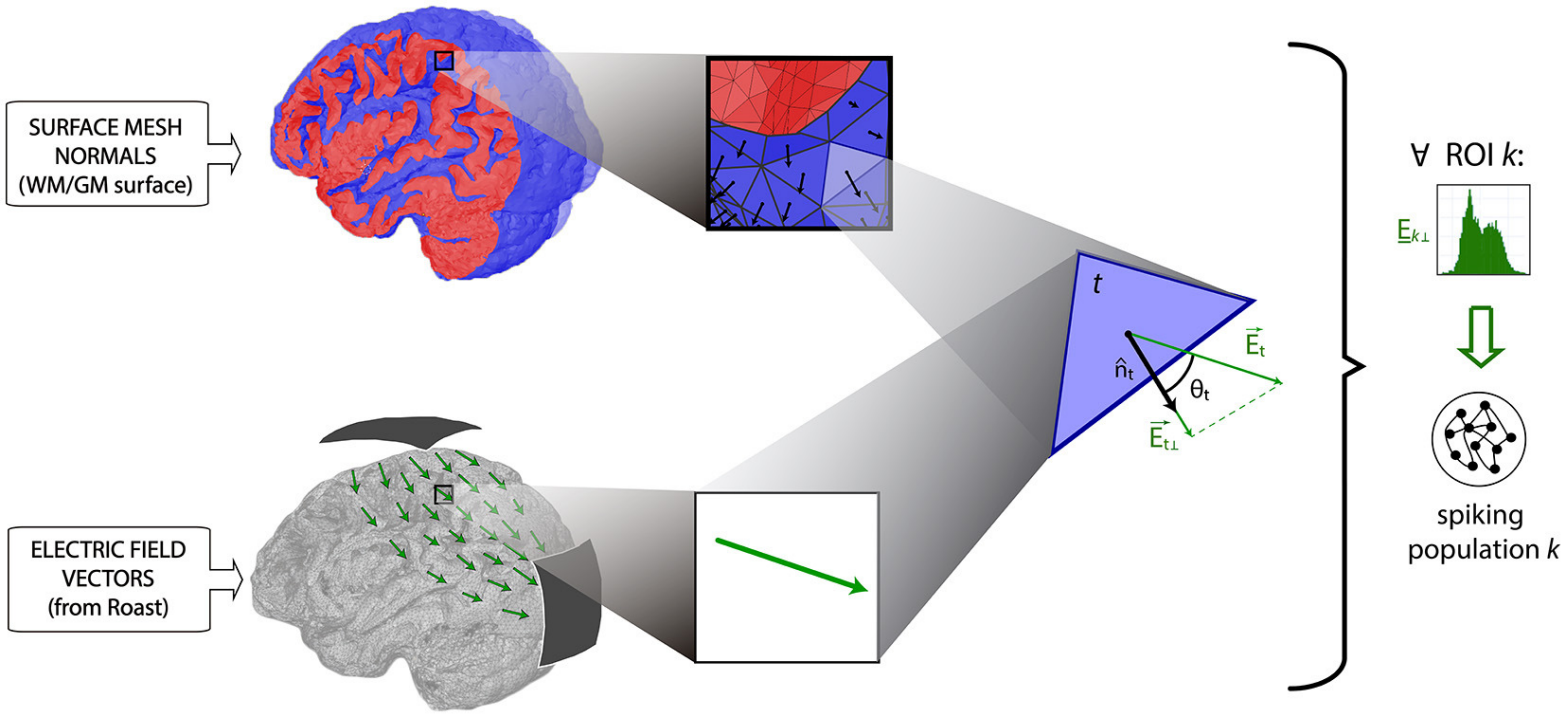
Estimation of the normal component of the electric field on triangle t of the white matter surface (*E*_*t*⊥_), as the dot product between the electric field in this point (i.e., $\vec{E_{t}}$, computed by Roast), and the normal vector to the surface t (i.e., $\hat{n_{t}}$, obtained from the triangular mesh). Note that $\hat{n_{t}}$ represents the direction of the cortical columns subtended to the cortex. Red color indicates a sagittal section of the gray matter, where blue color indicates its inner and outer surfaces. ROI-specific distributions of the normal components of E (i.e., *E*_*k*⊥_) are finally used for the stimulation of spiking populations.

Additionally, we took into account the orientation of the pyramidal cells' body axis by using an isometric triangular mesh of the boundary surface between white and gray matter. In this way, the value of the projections of the electric field to the normal direction referred to each surface triangle *t* (i.e., the normal component of $\vec{E}$ with respect to the triangle *t*, *E*_*t*⊥_) were computed through the scalar product:


(4) $$\begin{array}{l} E_{t⊥}=\vec{E_{t}}\cdot \hat{n_{t}}=|E_{t}|\cos\theta_{t} \end{array}$$

where $\hat{n_{t}}$ is the unit vector perpendicular to triangle *t* (see Figure 2) pointing toward the white matter volume. In this way, fields aligned with the orthodromic direction (dendritic tuft to axon) will result in positive values, as opposed to those aligned with the antidromic direction (Bikson et al., 2004; Merlet et al., 2013). For each subject, the projections of the electric fields were grouped for each region k of the AAL, to generate a set of distributions *E*_*k*⊥_ (see Supplementary Figure 2).

Finally, to implement the tACS within the SNN model, each region-specific distribution was used to generate a set of sinusoidal currents with heterogeneous amplitudes and fixed frequency. Defining a global calibration constant V for scaling the stimulation intensity, the current input for neuron *i* belonging to node *k* is:


(5) $$\begin{array}{l} I_{ext,i}^{k}\left(t\right)=A_{i}^{k}V\sin\left(2\pi ft\right) \end{array}$$

where $A_{i}^{k}$ is a random value obtained from the distribution related to *k*.

#### 2.2.5. tACS simulations

Two types of tACS simulations were carried out in this study with SNNs. First, we performed single-node simulations to test the effects of different hypothetical shapes on the distribution of normal components in the underlying neural activity. We explored the effect of the stimulation frequency (from 4 to 18 Hz), and amplitude (from 0 to 200 pA) over the underlying nodes' dynamics. To characterize the impact of the stimulation, we computed the power spectral density (PSD) of the simulated population extracting information regarding the frequency peak of the nodes, and the value of power at both the peak and the stimulation frequency. Second, we performed a calibration procedure to find the scaling factor V that maximizes the matching with the empirical observations. These simulations were performed considering the cingulum bundle networks (see *Spiking population model* section) of the ten subjects included in the computational dataset. Both types of simulations were performed 3 times for 50 s duration, removing the initial 4 s of potential transients.

#### 2.2.6. Statistics

We checked for statistically significant differences between the baseline simulations and the fitted using Wilcoxon's ranked comparisons (Wilcoxon, 1945) due to the small sample size, and correcting significance with a step-down method using Bonferroni adjustments (Holm, 1979).

To evaluate the impact of different variables on the increase of alpha power, we performed a stepwise multiple linear regression. We considered seven candidate variables per region: (*i*) the electric field modeled through the distributions of normal components; (*ii*) the squared value of the mean (as the effect is expected to be equivalent for negative and positive values and to linearize the data); (*iii*) the *skewness*, (*iv*) the *kurtosis*, and (*v*) the number of *modes*; (*vi*) the structural connectivity, with the logarithm of the nodes' *connectivity strength* to linearize the exponential shape observed in the structural connectivity data; and (*vii*) the network function before stimulation, using the average PLV value, and the frequency difference between the stimulation and the node's baseline oscillation. Two variables were transformed to get a better fit for the data. We used the square of the mean of the normal components of the electric field with the purpose of obtaining. Additionally, we used the base 2 logarithm of the node strength to get a better fit to the structural connectivity data.

The values of these variables were normalized in order to get a meaningful comparison of the resulting coefficients. Due to the violation of residuals' normality, we used an iteratively reweighted least squares algorithm as a robust version of the multiple linear regression. The weighted function for residuals was a Huber's T.

## 3. Results

### 3.1. Empirical results

#### 3.1.1. Participant demographics

From the 27 participants, six participants were discarded (three *verum* and three *sham*), due to the impossibility to achieve a proper impedance value for the neurostimulation session (*n* = 2), an increased wait time between the end of the stimulation and the beginning of the post recording (*n* = 2), or the impossibility to identify the participants' IAF (*n* = 2). The demographics of the participants meeting all the inclusion criteria can be found in Table 2.

**Table 2:** Demographics table for the empirical data.

	***Verum***	***Sham***	***p-value***
***N***	11	10	–
Age	35 ± 8.44	32.1 ± 9.37	0.273
Sex	8(M)/3(F)	5(M)/5(F)	0.387
IAF(Hz)	10.34 ± 1.09	10.23 ± 1.16	1

#### 3.1.2. IAF-tACS did not modulate occipito-parietal activity

While we expected an increase in power over the parieto-occipital areas after the stimulation, no significant differences were observed when comparing the rate of change in power between the *verum* and *sham* groups (*p* = 0.1216).

#### 3.1.3. The stimulation sustained the decay of alpha power in time

**Figure 3:**
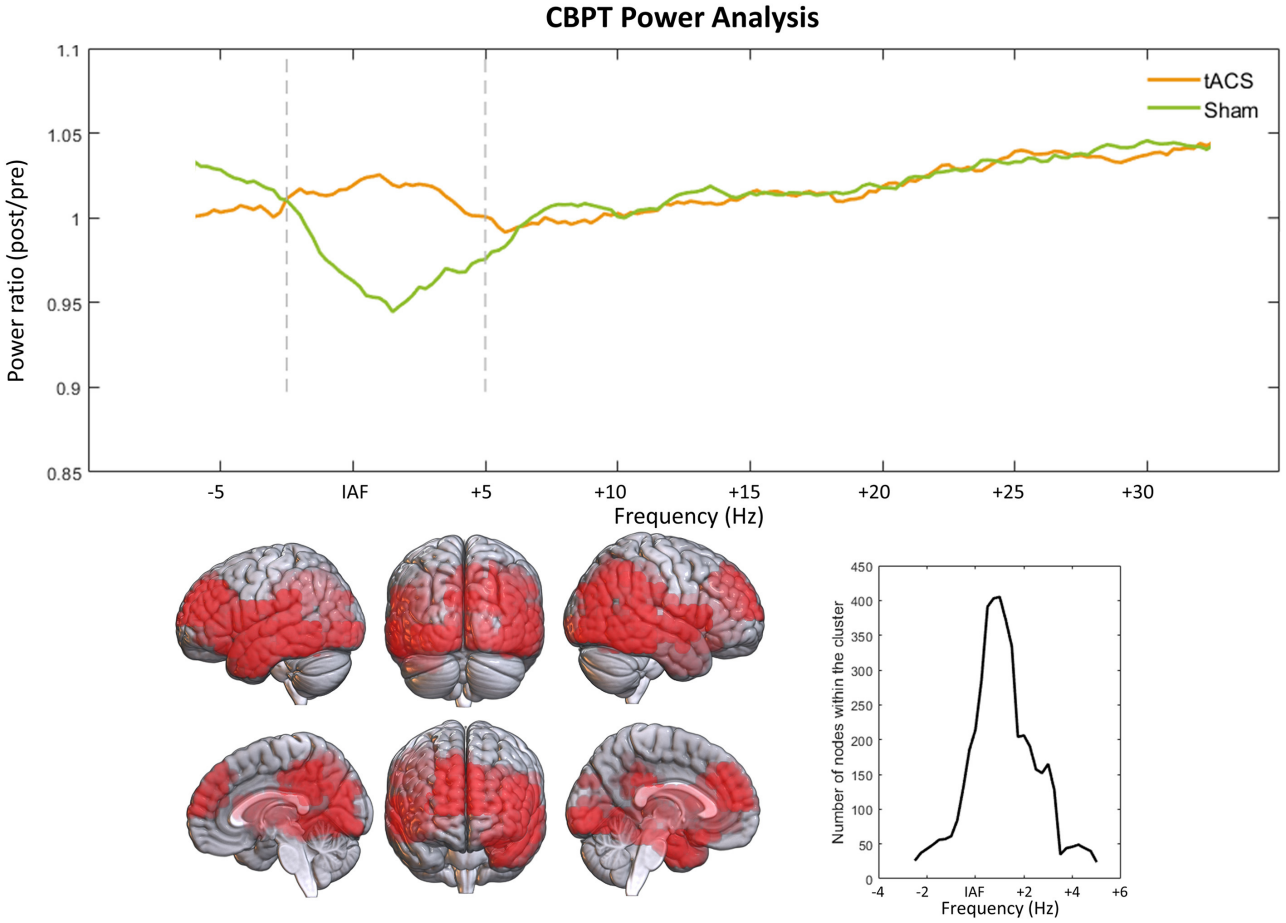
Power ratio comparison results through CBPT. Upper graph shows the power ratio (power from the post session divided by that of the pre2 session) over the whole spectrum. Vertical lines delimit the frequency range where the significant cluster was found (IAF − 2.5 Hz, IAF + 5 Hz). The lower part of the figure shows the distribution of the significant cluster over the brain, and the number of nodes included in the cluster over its different frequencies.

After performing CBPT analysis, it was found in the *verum* group a higher rate of change in power for frequencies between IAF−2.5 Hz and IAF + 5 Hz, with an increase in the power ratio of 8.02% in the *verum* group, in a cluster comprised of bilateral frontal, temporal and occipital cortical sources (*p* < 0.01; Figure 3). At low frequencies, the cluster is located in the inferior frontal gyrus and left temporal pole, spreading to fronto-orbital and temporo-occipital regions as the frequency increases. Although there was a significant increase in power observed in the *verum* group compared to the sham group, Figure 3 reveals that the stimulation prevented the decrease in power seen in the sham group. It is noteworthy that even though the stimulation targeted the IAF, the significant effects of the stimulation were observed across a broader frequency band, with the maximum number of significant nodes detected at IAF + 1 Hz.

#### 3.1.4. Neurostimulation effects are highly variable

**Figure 4:**
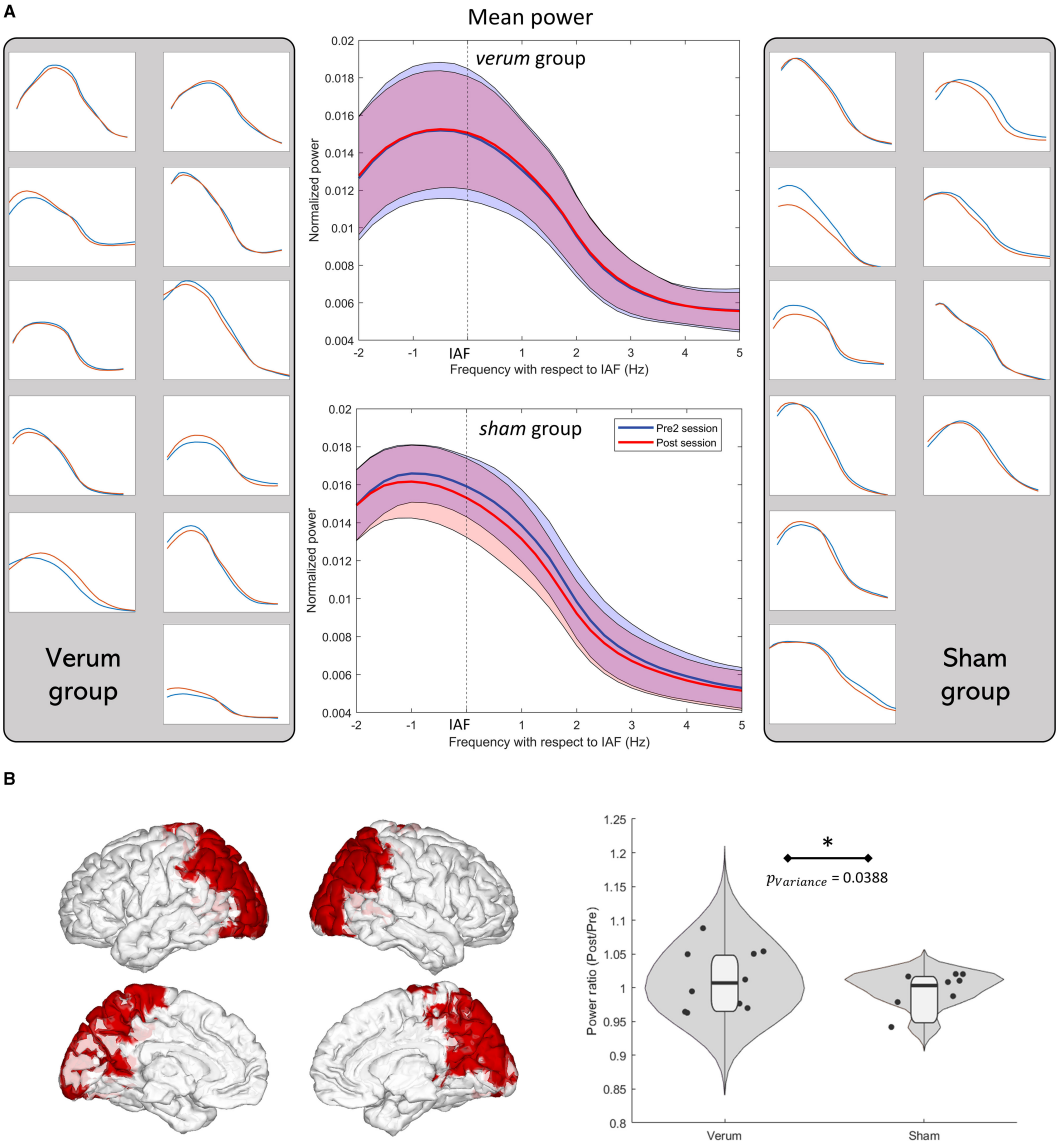
**(A)** Power spectra in the pre2 (curves in blue) and post (curves in red) sessions of all participants in the significant cluster presented in Figure 3 bottom-right panel. Verum participants are shown on the left and sham participants on the right. Mean power spectra of each group is shown in the middle. All graphs have the same axis scale. **(B)** Parieto-occipital regions of the AAL atlas where the variance in the verum group was significantly higher as well as the violin plots of the average power ratio in the same regions, both in the verum and sham groups. The violin plots were created using the RST toolbox for MATLAB (Pernet et al., 2013). Levene test showed that the verum group had a significantly larger variance than that of the sham group (*p* = 0.0388).

A *post-hoc* Levene test was conducted in the frequency band where the CBPT was found significant in the previous section (IAF−2.5 Hz, IAF + 5 Hz). The average power ratio in the parieto-occipital nodes of the brain was used, based on the AAL atlas (Figure 4B), where no significant changes in power were initially observed. The *verum* group showed a significantly larger variance (*p* = 0.0388) compared to the *sham* group, as shown in Figure 4B. Interestingly, there was no significant increase in the power ratio in this same region and frequency band (*p* = 0.1956). Figure 4A displays the individual spectra of all participants and the mean spectra of the *verum* and *sham* groups in the *pre2* and *post* sessions. These spectra were obtained in the previously described CBPT cluster. The graph shows the power depression in the sham group and the inconsistency of the effect of tACS in the *verum* group, with individual spectra showing both increases and decreases in the power rate.

### 3.2. Simulation results

#### 3.2.1. Cortical gyrification modulates the effects of tACS: evidence of contradictory outcomes

In order to perform the simulations, we calculated the electric field generated by the Oz-Cz stimulation protocol over the ten subjects in the computational sample. Given that the impact of tACS currents is primarily on pyramidal cells, and that the relative mismatch between the electric field direction and cell body axis can influence the efficacy of the stimulation, we computed the normal component of the electric field in relation to the white matter surface (see Section Methods). We grouped the components into regions of the AAL and then analyzed the results for the regions of the cingulum bundle.

**Figure 5:**
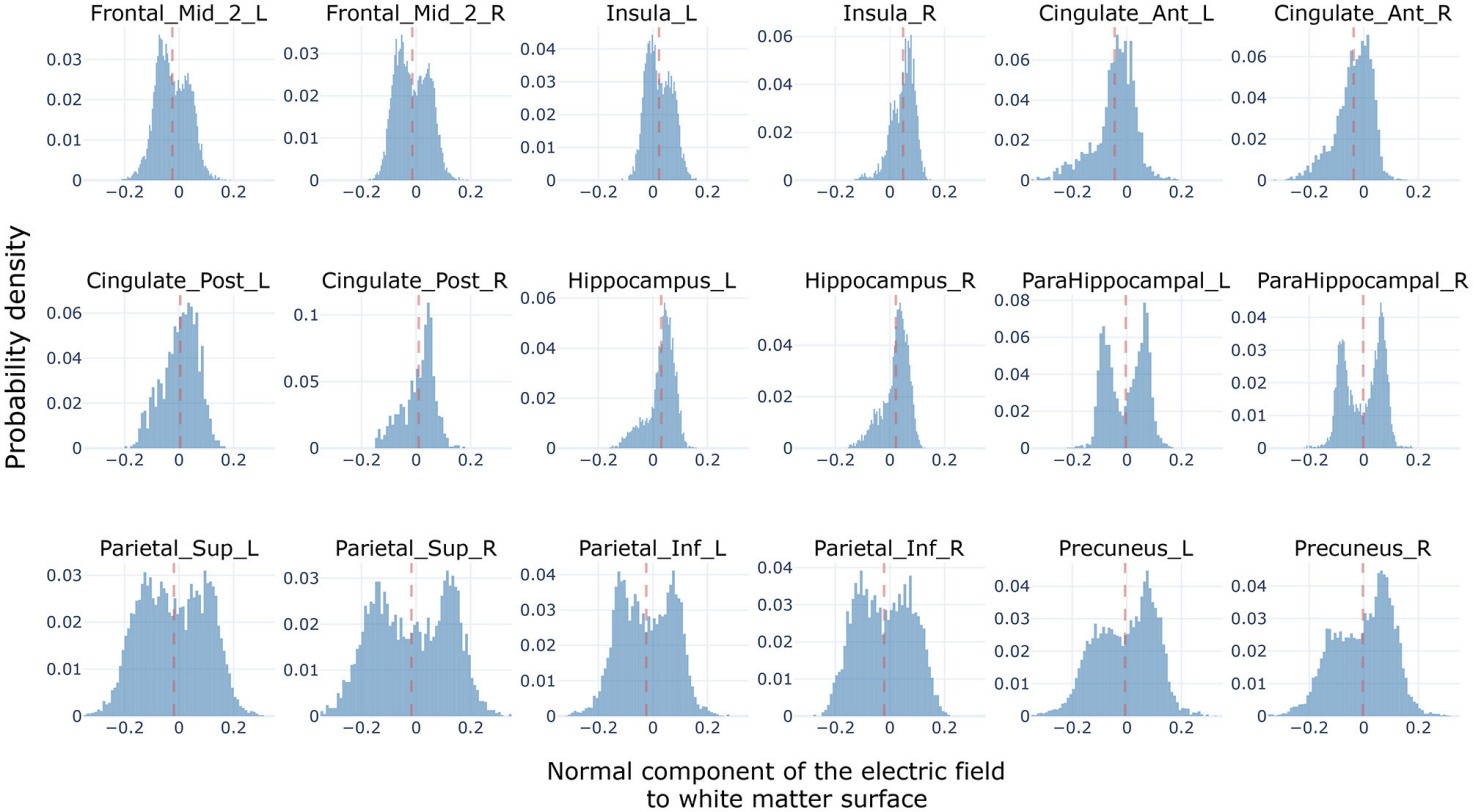
Normal component distributions (i.e., *E*_*k*⊥_). Histograms showing the accumulated (across subjects) distribution of normal components—to the white matter surface—of the electric field generated by the Oz-Cz stimulation protocol over the regions of the cingulum bundle, and including all subjects in the computational sample. Electric field distributions of subcortical regions are not included in the figure.

The Oz-Cz stimulation protocol induced currents flowing in the direction of the anterior-posterior brain axes (see Figure 1 in Methods). The evaluation of the direction of $\vec{E}$ with respect to the normal vectors of the triangulated surface between white and gray matter, interestingly showed two types of distributions (Figure 5): unimodal and bimodal distributions, according to the position and shape of different brain regions. All regions showed positive (i.e., oriented toward white matter) and negative values of *E*_*t*⊥_, meaning that all regions had at least some pyramidal cells oriented parallel and some oriented antiparallel to the electric field.

Regions situated along the antero-posterior axis, which are aligned with the orientation of the electric field such as the cingulate cortex, insula, and middle frontal gyrus, showed distributions that tended to be unimodal with a slight skewness, shifting the mean toward either positive or negative values. We found that the level of gyrification was related to the strength of the bimodality observed. For instance, the cingulate cortex, which is defined in the interhemispheric face of the brain, displayed less bimodality than the middle frontal gyrus which tends to have a more intricate shape. Interestingly, all these regions exhibited a bias toward the same value in both hemispheres. For instance, both left and right insulas were positively skewed, anterior cingulate cortices were negatively skewed, and both middle frontal gyri were negatively skewed.

Bimodal distributions were observed in posterior regions such as the parietal cortices and the precuneus, where the intricacy of the gyrification is maximized. These parietal regions have highly symmetric distributions around zero with two strong peaks, one positive and another negative. This implies that the gyri of these regions are mostly defined perpendicular to the orientation of the electric field. Therefore, it could be expected that by stimulating with the Oz-Cz protocol, certain cells in these regions get hyperpolarized, while others get depolarized. Although some studies (Aberra et al., 2020) have started to unravel the contradictory effects that an electric field might deliver to the pyramidal cells into a gyrus, it is yet unknown how these regions would interact with others inside a network.

#### 3.2.2. The distribution of normal field components modulates the effects of the stimulation

**Figure 6:**
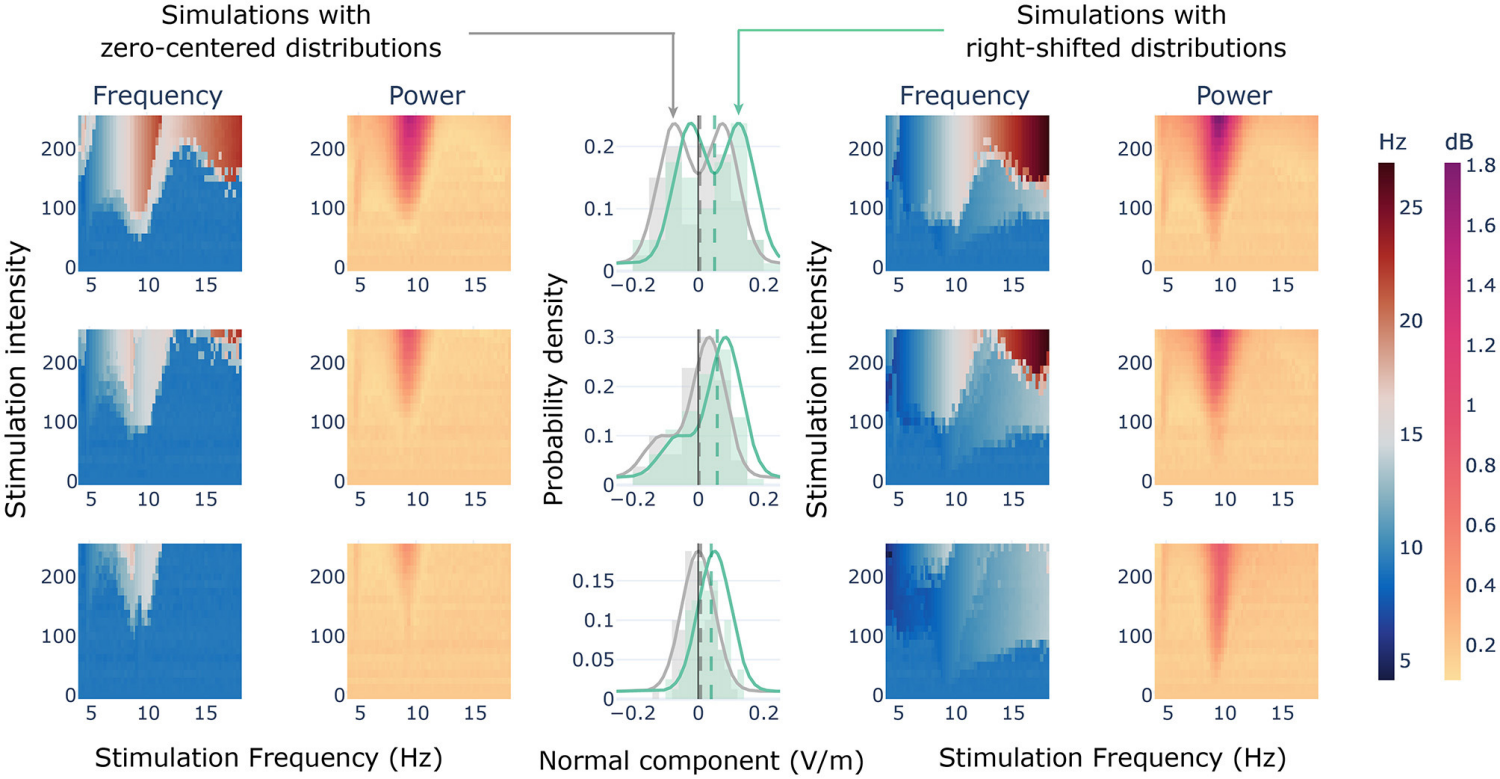
Stimulation of a single node with theoretical distributions. Central column showing the probability density distributions (i.e., curves) and the actual values extracted for the simulations (i.e., histograms). Vertical dashed lines showing the means of the values extracted for the simulations. In gray, the distributions with a theoretical mean centered at 0; in green, the right shifted distributions with a theoretical mean at 0.05. Lateral heat maps showing the results of simulating a single spiking node with the values extracted from the theoretical distributions within a range of frequencies and stimulation intensities.

We utilized spiking neural models to gain insights into the dynamics of a single population of neurons when exposed to two sets of anti-phase sinusoidal signals with varying amplitude values, as derived from the histograms presented earlier. To accomplish this, we defined a set of distributions that could illustrate the typical shapes observed in the regions of the cingulum bundle. We defined three pairs of distributions with different shapes: bimodal symmetric, bimodal asymmetric, and Gaussian. We centered one version of these prototypical distributions at mean 0, and another version at mean 0.05 (see Figure 6, center column). By treating those distributions as probability densities, we assigned an electric field component to each neuron in the network. Then, we simulated the activity of a node being stimulated with the assigned electric field components, for a defined range of frequencies and stimulation intensities (the *space parameter*).

In general terms, the results revealed that the mean of the distribution was not enough to capture the effects of the stimulation at the target node. For the distributions with zero mean, we observed that whenever entrainment was reached (1:1 synchronization state), the resulting oscillatory frequency of the node raised above the frequency of stimulation (Figure 6, right columns). Two levels of this behavior were found in the parameter space. With enough stimulation intensity, and frequencies close to the node's natural frequency of oscillation, we found a region in which the stimulation produced an oscillatory dynamic at twice the stimulation frequency (2:1 synchronization state). This region was wider for the symmetric bimodal distribution than for the other two (Figure 6, top left). Indeed, for the asymmetric bimodal and the Gaussian (Figure 6, center and bottom left, respectively), the 2:1 synchronization effect was found just for stimulation frequencies lower than the node's own oscillatory dynamic. This 2:1 response is due to the opposite and alternating polarizing effects that the stimulation exerts over the neurons, some of them were receiving a depolarizing current, while others were receiving a hyperpolarizing current. Therefore, in one period of the tACS wave two different sets of neurons become depolarized, generating neuronal discharges at a doubled oscillatory frequency. Surrounding this 2:1 response, we found a region of the parameter space in which the induced dynamics were ~50% faster than the stimulation. In terms of power, the highest increases were found at the natural frequency of the node. Additionally, a slight decrease could be observed at the borders of the doubled-frequency Arnold's tongue.

For the positively shifted distributions (Figure 6, right columns), we found the emergence of the classical Arnold's tongues, in which the frequency of stimulation equals the frequency of the node for enough stimulation intensity (1:1 synchronization states). Also, the 2:1 synchronization state region of the parameter space mentioned previously was found for these distributions, as well as an additional region with an ~20% slower dynamic than the stimulation frequency, that expands from the nodes' frequency to higher frequencies with lower stimulation intensity threshold.

After comparing the three pairs of distributions, we have reached a conclusion that although they had the same mean, the bimodal symmetric distribution had more intense entrainment and increase in power than the bimodal asymmetric and Gaussian distributions. Hence, the probability distribution shape, in addition to the external frequency and stimulation intensity, plays an essential role in the emergence of different dynamical states discussed earlier.

#### 3.2.3. The mean of the distribution is the main predictor of alpha power rise

To investigate the sources of variability observed in the empirical results, we constructed a spiking neural network model consisting of 22 regions of interest using the data from our computational dataset. We then implemented a tACS stimulation protocol using Oz-Cz pad electrodes. We calculated the distributions of normal components per subject and used them as probability density distributions to assign a component to each neuron. In this section, we calibrated the impact of tACS currents over the regional dynamics of our models (i.e., stimulation intensity) by fitting the calibration constant V to a value in which a group-averaged rise of 8.02% in alpha power is found in the same cluster of regions that emerged from the empirical experiment. Once calibrated, we used a robust multiple linear regression model to evaluate the impact of different variables in the power rise.

**Figure 7:**
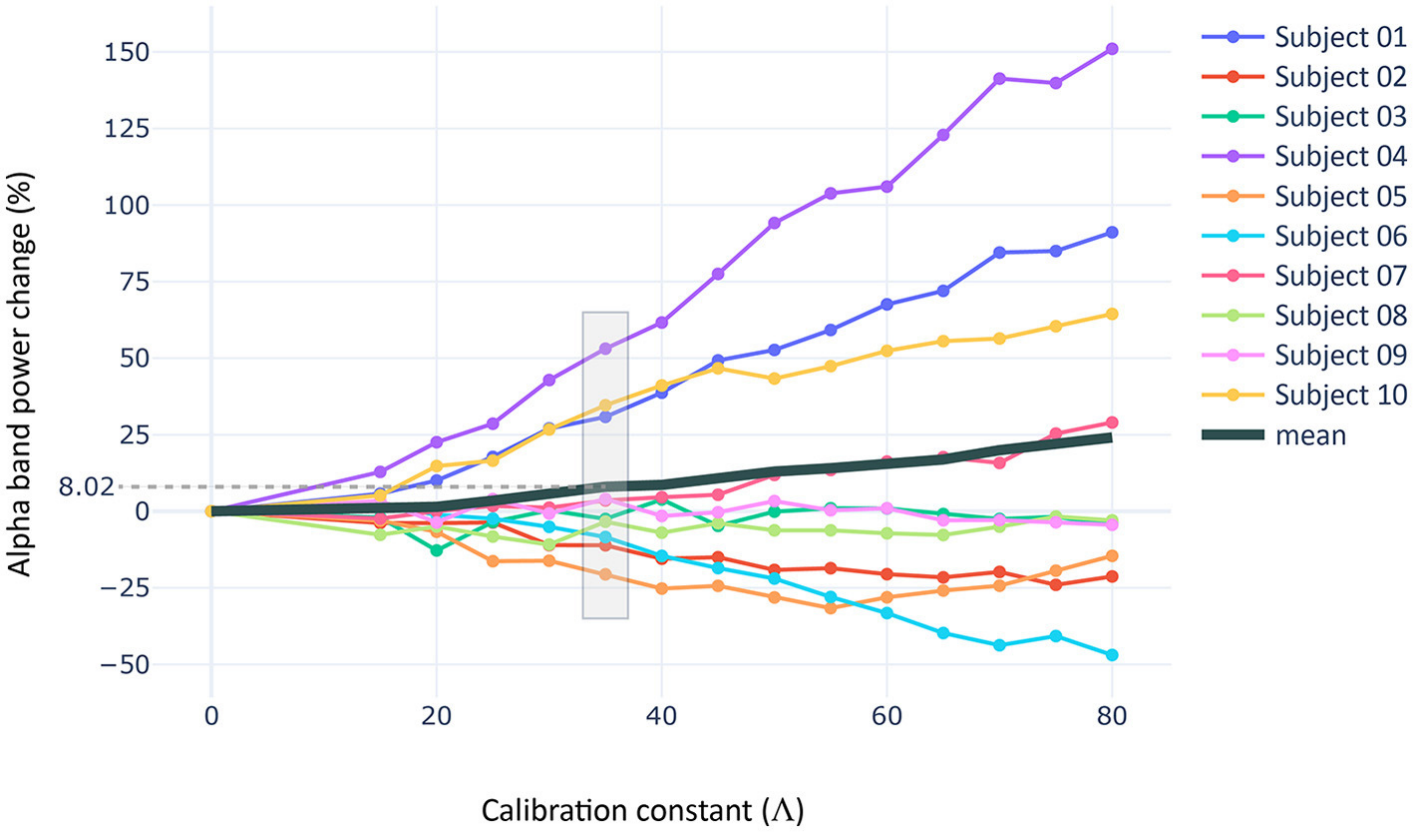
Stimulation intensity fitting. Procedure used to fit the intensity of stimulation to the results of the empirical experiment. The scatter shows the percentage of alpha band power rise for thirty simulations per intensity value and subject in the computational sample. The alpha band power rise is measured in the regions of the CBPT cluster described in the empirical section, and considering a frequency band (± 0.5 Hz) around the trial-specific IAF measured in our models.

The calibration process, similar to empirical results, revealed a variable effect of the stimulation on the subjects included in the computational sample (Figure 7). We found a fit for *V* = 35 (see Supplementary Figure 3 to observe the detailed effect on each region, Supplementary Figure 4 for the simulated LFPs during baseline and stimulation stages, and Supplementary Figure 5 for single node dynamics). At this level of intensity, three subjects increased significantly their alpha power from baseline [~40% rise; W = 0, *p* < 0.05, RBC = 1, CLES = 0.85], while the rest of the sample did not exhibit statistically significant changes (i.e., alpha power fluctuated around zero). However, moderate reductions in alpha power were observed among these subjects (~10−−25% decrease).

**Figure 8:**
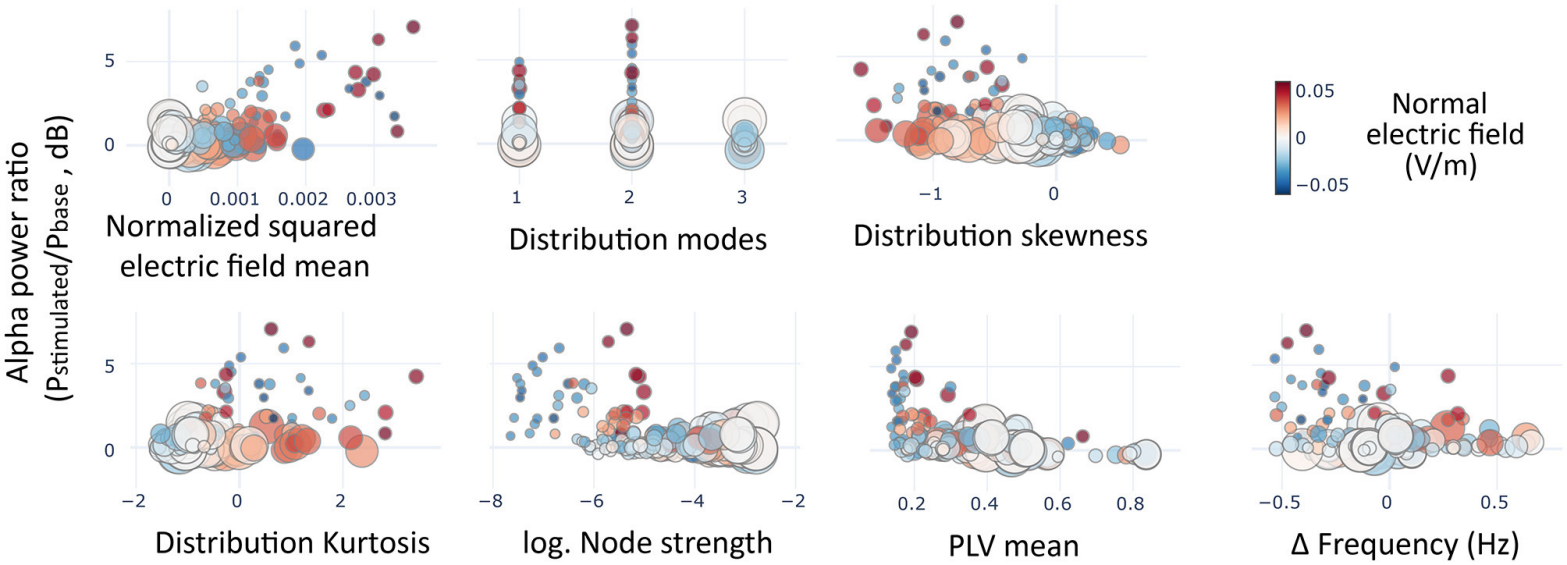
Predictors of alpha rise. Scatter plots showing the average alpha power variation in the simulated regions (circles) as a function of the variables included in the multiple linear regression model. In size, the mean node strength of the region, and in color the average of normal components of the electric fields pertaining to each region.

With the calibrated computational model, we performed a stepwise multiple linear regression (MLR) to evaluate the impact of different variables in the increase of alpha power for the regions included in the model. We considered seven candidate variables regarding structural connectivity, electric field modeling, and network function before stimulation (see Figure 8). The values of these variables were standardized in order to get a meaningful comparison of the resulting coefficients. One variable was discarded during the stepwise process due to non-significance: the difference between the stimulation frequency and the baseline frequency of the node (coefficient = 0.043, SE = 0.037, *p* = 0.236). The rest of the variables showed statistically significant coefficients. The most relevant coefficient was related to the mean of the distribution of the electric field's normal component that was directly related to the change in alpha power (coefficient = 0.44, SE = 0.039, *p* < 0.0001). Other measures describing the distribution had also statistically significant coefficients, including the skewness (coefficient = −0.256, SE = 0.049, *p* < 0.0001), kurtosis (coefficient=-0.137, SE=0.053, p=0.009), and the number of modes (coefficient = −0.0687, SE = 0.029, *p* = 0.017). Additionally, regarding connectivity variables the functional connectivity of a region previously to the stimulation was a better predictor of alpha rise (coefficient = −0.2, SE = 0.034, *p* < 0.0001) than the structural connectivity (coefficient=-0.1, SE=0.037, *p*=0.008).

From the model coefficients, it could be derived that all the significant variables, except the squared mean of the distribution, could be responsible for the slight lowering of alpha power in certain simulated subjects (e.g., subjects five to seven) as negative coefficients. Given the relatively small magnitude of alpha lowering with respect to the rise, we wanted to discern more clearly whether those variables were directly related to a reduction in power or whether they were involved in softening a rising effect. To do this, we performed an additional MLR model using just the data from regions whose power lowered with the stimulation (see Supplementary Figure 6). This model showed that only the functional connectivity (coefficient = −0.0423, SE = 0.007, *p* < 0.0001) could predict the lowering in power, with the protective effect of structural connectivity (coefficient = 0.024, SE = 0.01, *p* = 0.015). All other candidate variables did not reach significance.

## 4. Discussion

Non-invasive brain stimulation has been proposed as a candidate tool for the treatment of brain disorders, and specifically, tACS has shown the potential to interact and modulate endogenous rhythms shaping brain dynamics and cognition. However, the mechanisms underlying the effects of this technique remain elusive. In this study, we replicated an IAF-tACS stimulation protocol with square patches at Oz-Cz positions, intending to rise alpha power in occipito-parietal regions. Additionally, we used computational modeling to dig into the mechanisms that might be mediating the effectiveness of its application. We calculated a current propagation model through head tissues into the brain and extracted the components of the electric field that are orthogonal to the white matter surface (i.e., the normal component of the electric field) to take into account the effect of the electric field orientation. Finally, we used this information to build an SNN model in which we could systematically test different aspects of brain activity under stimulation. This study provides a deeper understanding of the variables affecting brain dynamics under stimulation.

Our empirical results were in line with previous research findings showing an increase in power that involved many brain regions, with the exception of some occipito-parietal ones (Kasten et al., 2019). This is an unexpected finding, opposite to what we hypothesized, as the stimulation was delivered over occipito-parietal regions, where the alpha power tends to be more prominent and the induced current density is maximized. At the same time, we observed an increased variability of the power change over the same areas in the verum group. Based on the results of our computational model, these unexpected findings could be related to the distributions of normal components of the electric field found in our study for parietal regions, as we hypothesized. In these cases, the reduced effect found in certain areas could potentially be explained by a bimodal distribution of the electric field with respect to the pyramidal neurons' body axis. This would result in a mean of *E*_*t*⊥_ close to zero, which our simulations showed to be the best predictor of power increase. These distributions might be particularly important to consider when translating tACS application results across different species (Beliaeva et al., 2021), as significant differences in cortical gyrification between species may confound the results. Other studies that have focused on analysing occipito-parietal regions have reported contradictory results (Zaehle et al., 2010; Zarubin et al., 2020), with some reporting increases in alpha power after tACS stimulation, while others report decreases.

Brain stimulation studies have revealed significant intersubject variability in the effects of applied protocols (Krause and Cohen Kadosh, 2014; Kasten et al., 2019; Wischnewski et al., 2023). This variability was also observed in both our empirical study and simulations. In the empirical study, we found subjects in the stimulation group that responded in three different ways: rising alpha power, getting the same power, and lowering it. Interestingly, a similar result was found in the simulations, in which three subjects raised significantly their alpha power while others kept the same value or lowered it. The MLR model, in combination with the distributions of normal components, could explain why some subjects exhibited enhancements in power while others did not. For instance, high functional or structural connectivity protects a region from entraining with the stimulation.

To explain why some subjects reduced alpha power, we performed a second MLR model focusing on lowering alpha-power values, and the best predictor was found to be the mean functional connectivity of a region: higher values of PLV predicted larger reductions. This is in line with previous research suggesting that the stimulation entrainment competes with the internal entrainment of the network between neighbors (Krause et al., 2022). Therefore, the stimulation could reduce the internal entrainment of the network, by modulating the regional oscillatory activity, and leading to a reduction in power.

An additional factor, not captured by the MLR models, may influence the rise/decay of the alpha power: the inter-regional communication through synaptic coupling. Previous research on information transmission in neural networks have suggested that communication between regions, in addition to the degree of coherence (PLV), depends on the conduction delay, determined by axon length, and the frequency mismatch between them (Pariz et al., 2021; Sánchez-Claros et al., 2021). The interplay of these two factors may enable or disable communication pathways trough regions. In a favorable scenario of effective communication, the power rise could be transmitted inter-regionally, and contribute to the power increase of connected regions (see Supplementary Figure 7). Consequently, it is reasonable to infer that from our neural network models may emerge subnetworks with optimized inter-regional communication that would benefit alpha band power rise. Nevertheless, a more comprehensive analysis is needed to investigate this possibility, thus paving the way for further research.

Optimizing the dosage and montages in brain stimulation is a current challenge that must be faced to achieve the desired effects from the intervention (Wischnewski et al., 2023). The complexity of this process is importantly limited by the stimulation hardware at use and implies the availability of structural images of the brains to be stimulated. However, in the process of optimization, it is often disregarded the orientation of the electric field with respect to brain tissue to focus on the maximization of the delivered field module and the spatial accuracy, despite the empirical and computational evidence that is raising awareness regarding the importance of this concept (Kabakov et al., 2012; Modolo et al., 2018; Aberra et al., 2020; Wang et al., 2023). In this study, we presented a simple way of employing the distributions of normal components, which could be integrated into the optimization protocols to take into consideration the orientation of the pyramidal cells' body axis in tACS.

The SNN simulations of theoretical distributions showed differential effects depending on the shape of the distributions of EF normal components while sharing approximately the same mean. In contrast, further regression analysis showed that although it does not explain the whole variance, the absolute mean of the distribution was the best predictor for alpha rise in a region. It should be noticed that the fitted value to empirical data of stimulation intensity used for regression was in the lower range of the theoretical experiments, in which the different results for distribution shapes were less prominent. This could suggest that using the mean of the distribution as in previous studies (Merlet et al., 2013) could be an acceptable approximation to model the effects on power, although missing a certain level of accuracy. Importantly, we assumed spatial homogeneity in the distribution of excitatory and inhibitory neurons in our SNN models, being this common practice in whole-brain modeling (Deco and Jirsa, 2012; Nakagawa et al., 2014; Stefanovski et al., 2019; Kazemi and Jamali, 2022). However, future studies should consider spatial inhomogeneity in their methodology to capture a higher degree of diversity in regional dynamics.

In conclusion, this study contributes to the understanding of the tACS mechanisms that modulate brain activity by combining empirical and computational approaches. We investigated the variables affecting brain dynamics under stimulation revealing unexpected findings. Additionally, the orientation of the electric field with respect to brain tissue was identified as a crucial factor in optimizing the dosage and montages for brain stimulation. This study rises awareness on the relevance of acquiring MRI data from the participants to effectively design the stimulation protocols. Furthermore, clinical trials involving this kind of technology as a treatment, such as those developed for depression, anxiety disorders or schizophrenia among others, could benefit from taking into account the direction of the elicited electric fiends in the brain in order to increase the likelihood of success of their neuromodulatory approaches. One limitation of our study is the fact that the empirical and computational datasets are different, as we did not have MRI scans of the participants that underwent neuromodulation. Thus, a further study combining empirical and computational approaches on the same sample of subjects would be of interest to confirm the observations made in this research. Non-invasive brain stimulation techniques, and specifically tACS, are potential tools for the treatment of brain disorders, however further research is necessary to fully understand and control the effects of these techniques on brain dynamics and cognition. Computational models would help in shaping stimulation protocols, providing a model driven approach for the application of tACS achieving more specific targets of brain signals and potentially improving results of neuromodulation.

## Data availability statement

The data supporting the conclusions of this article will be made available by the authors, without undue reservation. The clean data used in this study can be found in the following dropbox folder: https://www.dropbox.com/sh/nap4v19b390ptxz/AAC8IvWFs5JpAF-4LzRpUE_Oa?dl=0. Further inquiries can directed to the corresponding author.

## Ethics statement

The studies were conducted in accordance with the local legislation and institutional requirements. Informed consents were obtained from every participant before their participation.

## Author contributions

JS-C and JC-Á developed the software for simulations and analysis. MC-G and AC-L collected and analyzed empirical data. JC-Á and MC-G wrote the original draft of the manuscript. JS-C, AC-L, GS, FM, and CRM reviewed the manuscript. GS, CRM, and FM supervised the research and provided guidance for the theoretical framework. All authors contributed to the conceptualization. All authors contributed to the article and approved the submitted version.
