# Corrigendum: Understanding the effects of cortical gyrification in tACS: insights from experiments and computational models

**DOI:** 10.3389/fnins.2023.1329826

**Published:** 2023-11-17

**Authors:** Jesús Cabrera-Álvarez, Jaime Sánchez-Claros, Martín Carrasco-Gómez, Alberto del Cerro-León, Carlos J. Gómez-Ariza, Fernando Maestú, Claudio R. Mirasso, Gianluca Susi

**Affiliations:** ^1^Centre for Cognitive and Computational Neuroscience, Complutense University of Madrid, Madrid, Spain; ^2^Department of Experimental Psychology, Complutense University of Madrid, Madrid, Spain; ^3^Instituto de Física Interdisciplinar y Sistemas Complejos (IFISC, UIB-CSIC), Campus UIB, Palma de Mallorca, Spain; ^4^Biomedical Image Technologies, ETSI Telecomunicación, Universidad Politécnica de Madrid, Madrid, Spain; ^5^Department of Psychology, University of Jaén, Jaén, Spain; ^6^Department of Structure of Matter, Thermal Physics and Electronics, School of Physics, Complutense University of Madrid, Madrid, Spain

**Keywords:** tACS, spiking neural networks, brain network models, MEG, neuromodulation

In the published article, there is an error in the email of the corresponding author, Martín Carrasco-Gómez. Instead of martin.carrasco@ctb.upm.es the correct email address is martin.carrasco@upm.es.

In the published article, there was an error in the Funding statement. The declaration lacks reference to a research project. The correct Funding statement appears below.

## Funding

JC-Á and MC-G were funded by the Spanish Ministry of Universities through predoctoral FPU grants, references FPU2019-04251 and FPU2018-00517, respectively. JS-C and CM acknowledge support from the Spanish Ministerio de Ciencia e Innovación, Agencia Estatal de Investigación (PID2021-128158NB-C22/10.13039/501100011033) and Programs for Units of Excellence in R&D María de Maeztu (CEX2021-001164-M/10.13039/501100011033). FM and GS acknowledge funding by MCIN/AEI/10.13039/501100011033 and European Union (NextGenerationEU/PRTR) through the project PCI2021-122069-2A-Collaborative Research in Computational Neuroscience program.

The authors apologize for these errors and state that this does not change the scientific conclusions of the article in any way. The original article has been updated.

