# Supplementary material for: Understanding the effects of cortical gyrification in tACS: insights from experiments and computational models
Source: Front Neurosci. 2023 Aug 16;17:1223950. doi: 10.3389/fnins.2023.1223950 (PMC10467425; doi:10.3389/fnins.2023.1223950)
Supplement: Supplementary file 1 [file Data_Sheet_1.PDF]

Supplementary Material

1 SUPPLEMENTARY FIGURES

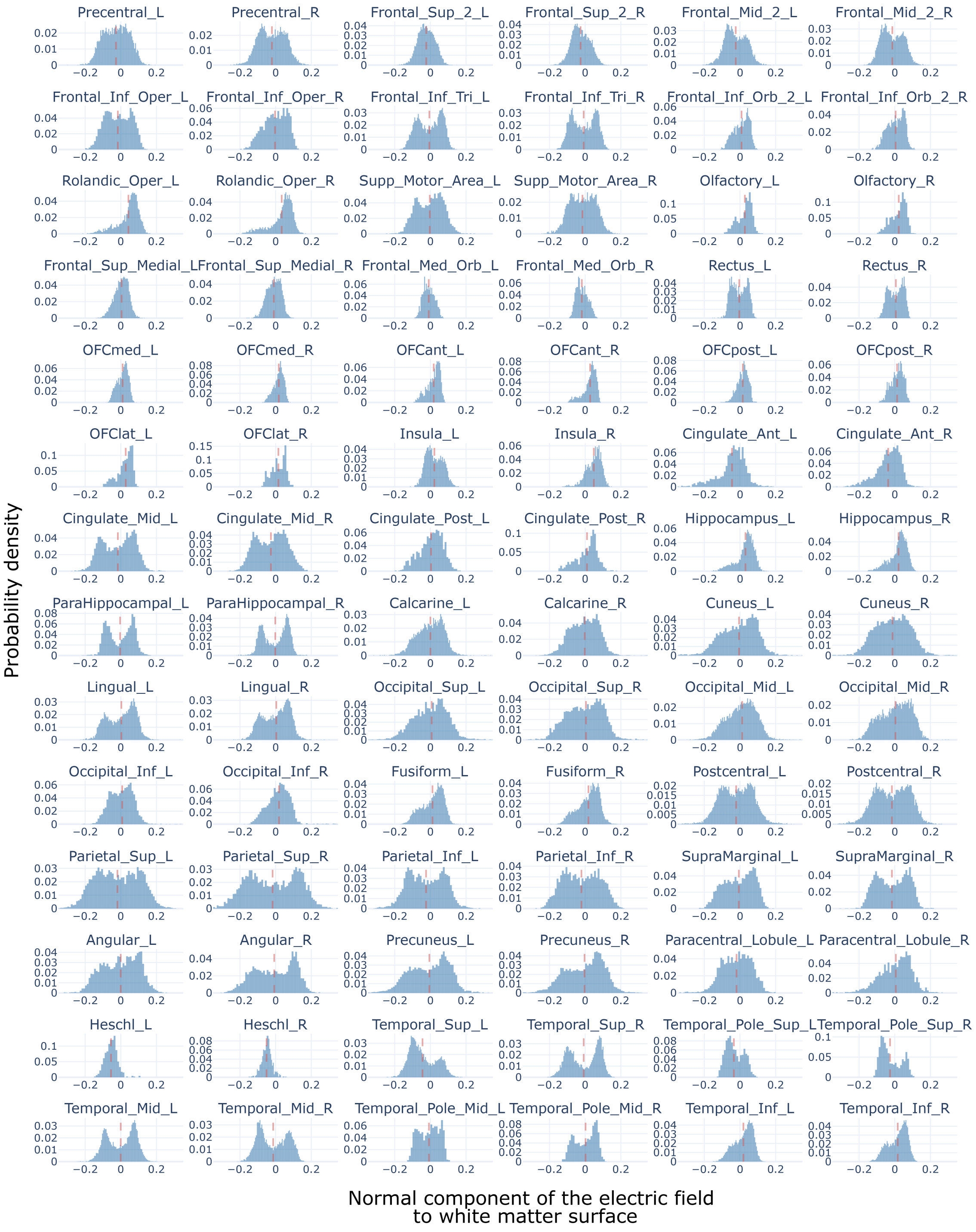

Figure S1. Distributions of the normal components in the cortical regions of AAL

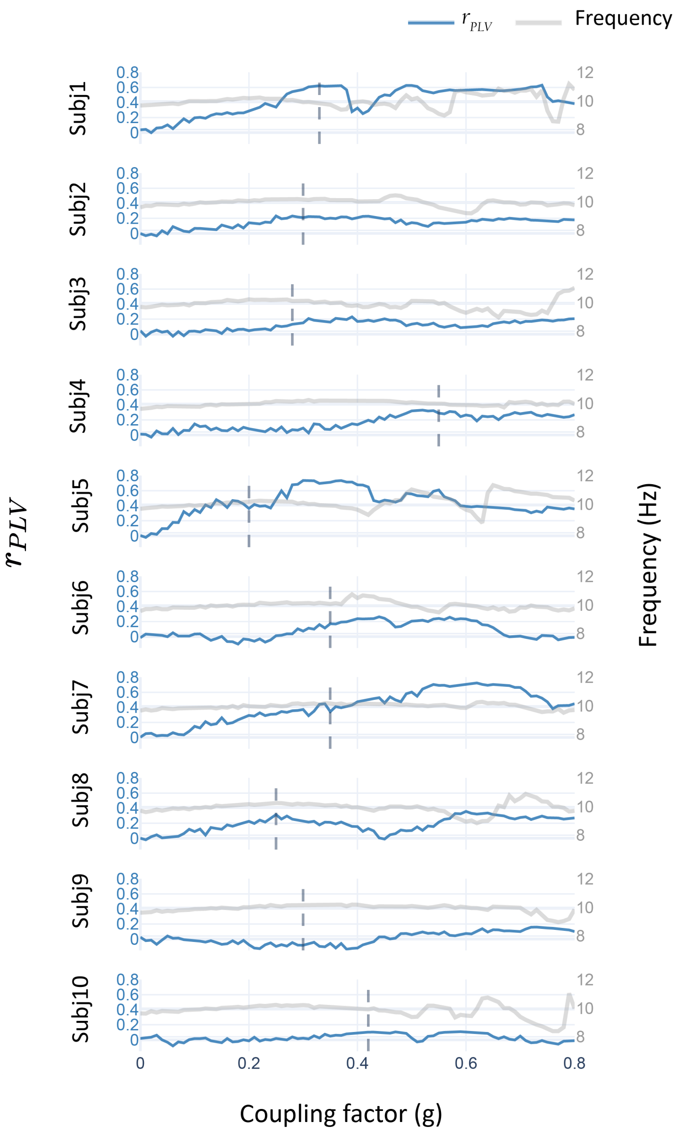

Figure S2. Working points for the subjects in the computational sample. Simulating each virtual brain network for a range of coupling factor values [0-0.8]. In blue, the correlation between empirical and simulated PLV. In gray, the mean frequency of oscillation. Vertical dashed line corresponds to the selected working point.

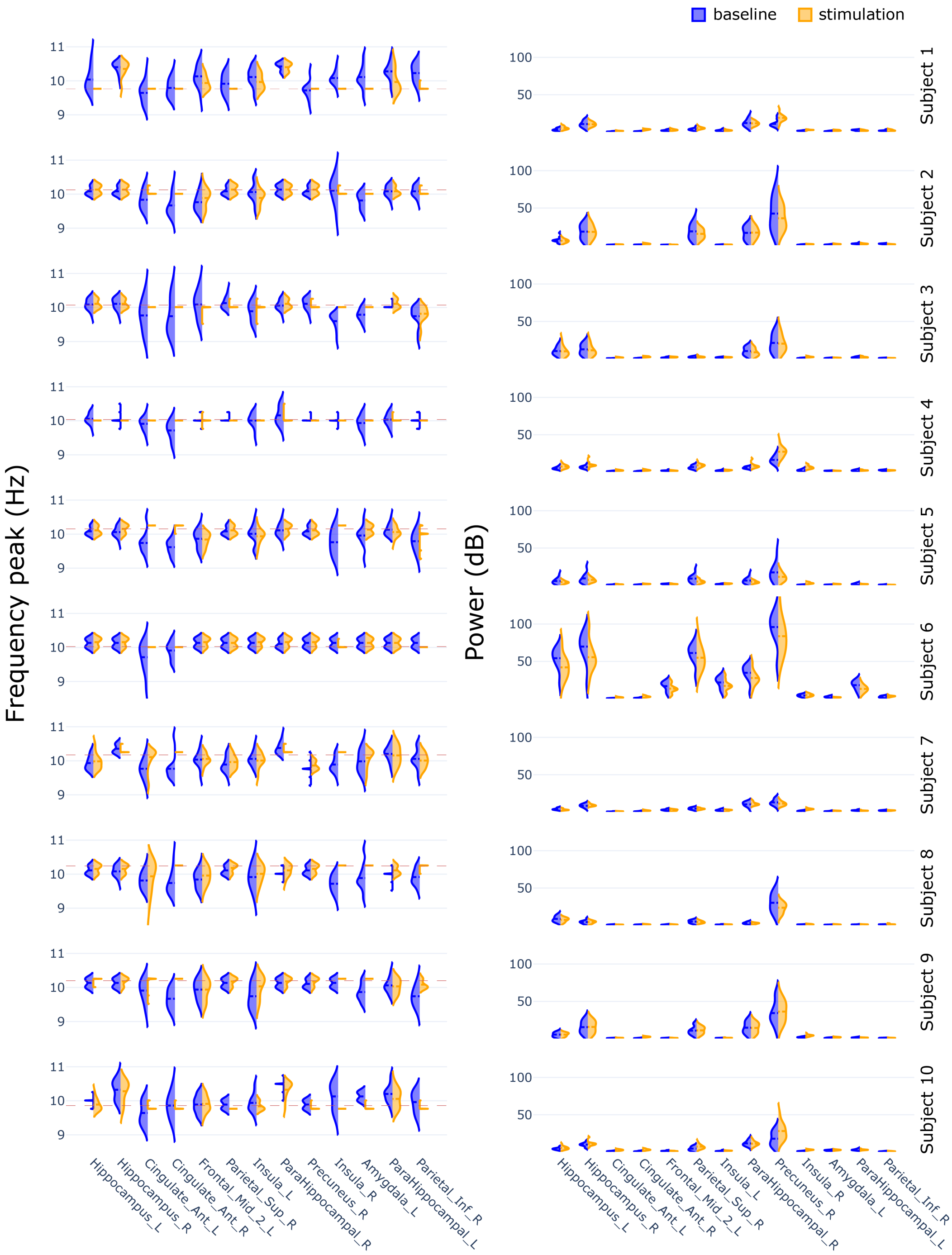

Figure S3. Results of the SNN simulations per node included in the CBPT cluster of alpha power rise regions. Showing the oscillatory frequency of each node before and during stimulation / pre and post stimulation when $\Lambda = 35$.

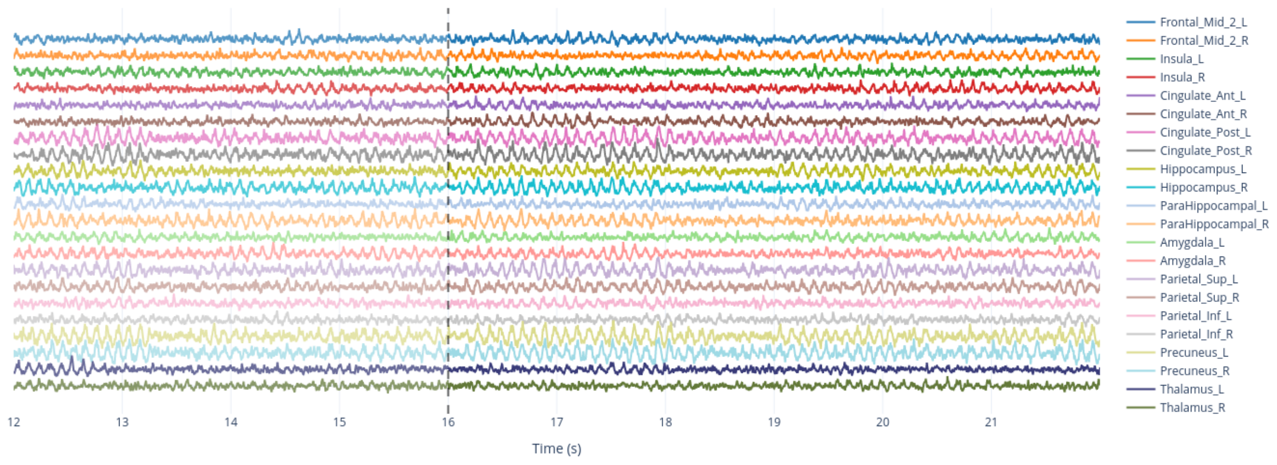

Figure S4. Simulated LFP timeseries for subject one. Vertical dashed line separates baseline (left) and stimulation stages (right).

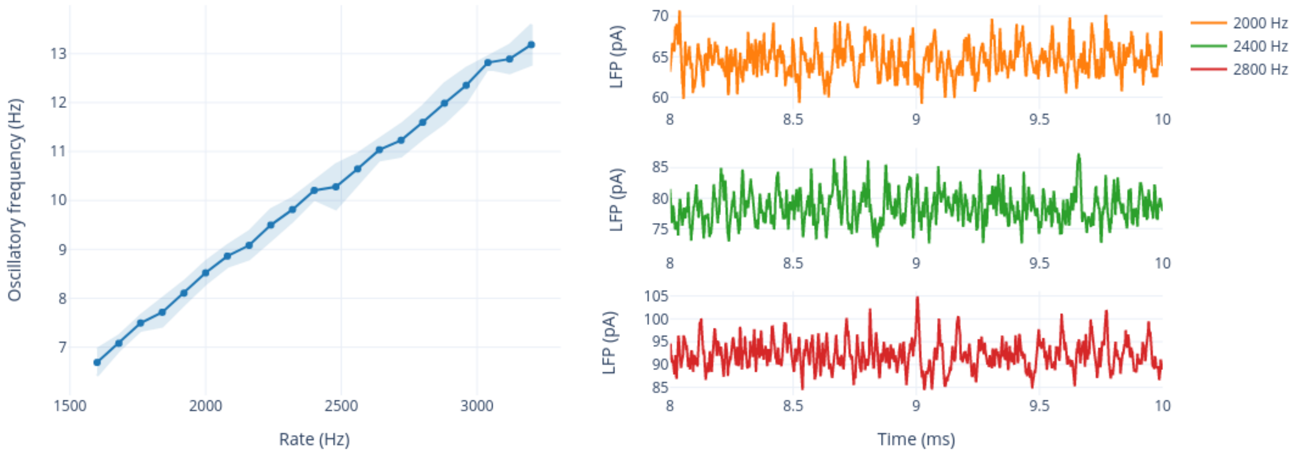

Figure S5. Single node dynamics. Left panel represents the frequency-current curve, where to control the intensity of the incoming current we used the rate of the Poissonian spike train that every neuron receives. Left panels show different LFPs for different values of the rate.

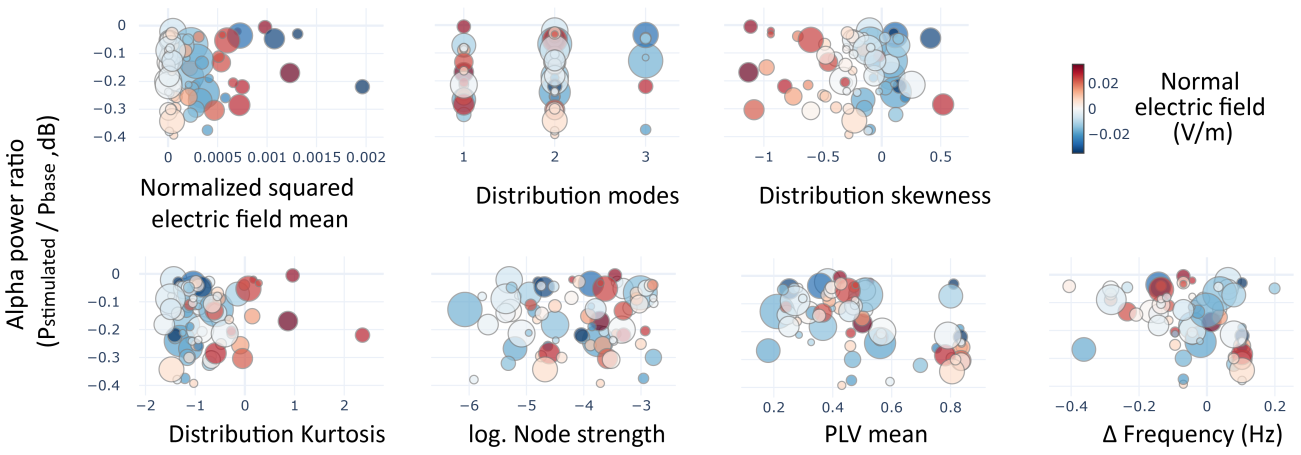

Figure S6. Predictors of alpha lowering in simulations. Scatter plots showing the average increase in alpha power in the simulated regions that lowered the power from baseline as a function of the variables included in the multiple linear regression model. In size, the mean node strength of the region, and in colour the normal component of the electric field. PLV mean was the only significant predictor of alpha power lowering.

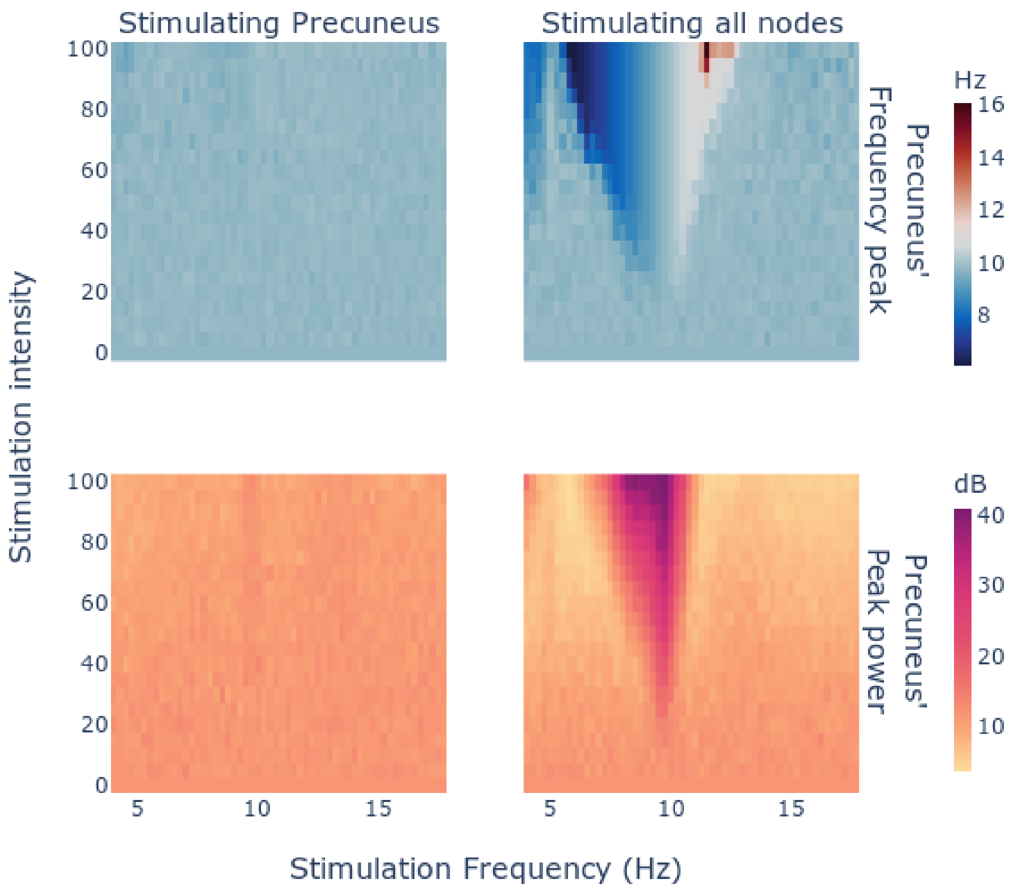

Figure S7. Frequency peak and its power in the Precuneus_R region of the network model when only this region is stimulated (left) and when the stimulation is over the whole network (right). This is an example of how the inter-regional synaptic transmission plays a role in the alpha rise power, since the direct stimulation does not produce an effect.
